# Supplementary material for: Axion insulator state in hundred-nanometer-thick magnetic topological insulator sandwich heterostructures
Source: Nat Commun. 2023 Nov 21;14:7596. doi: 10.1038/s41467-023-43474-x (PMC10663498; doi:10.1038/s41467-023-43474-x)
Supplement: Supplementary file 1 — Supplementary Information [file 41467_2023_43474_MOESM1_ESM.pdf]

Supplementary Information

Axion Insulator State in Hundred-Nanometer-Thick Magnetic Topological Insulator Sandwich Heterostructures

Deyi Zhuo[1,#], Zi-Jie Yan[1,#], Zi-Ting Sun[2,#], Ling-Jie Zhou[1], Yi-Fan Zhao[1], Ruoxi Zhang[1], Ruobing Mei[1], Hemian Yi[1], Ke Wang[3], Moses H. W. Chan[1], Chao-Xing Liu[1], K. T. Law[2], and Cui-Zu Chang[1]

[1] Department of Physics, The Pennsylvania State University, University Park, PA 16802, USA

[2]Department of Physics, Hong Kong University of Science and Technology, Clear Water Bay, 999077 Hong Kong, China

[3]Materials Research Institute, The Pennsylvania State University, University Park, PA 16802, USA

[#]These authors contributed equally.

Corresponding authors: cxc955@psu.edu (C.-Z. C.); phlaw@ust.hk (K. T. L.)

Content:

I. **Supplementary Figures**

II. **Supplementary Text**

1. **Gate-dependent transport measurements on the *m*=100 sample**
2. **The connection between the decay of the two-terminal resistance and the reduction of the transport gap**
3. **Discussion on the thickness limit of the axion insulator state in magnetic TI sandwiches**

Supplementary References

I. Supplementary Figures

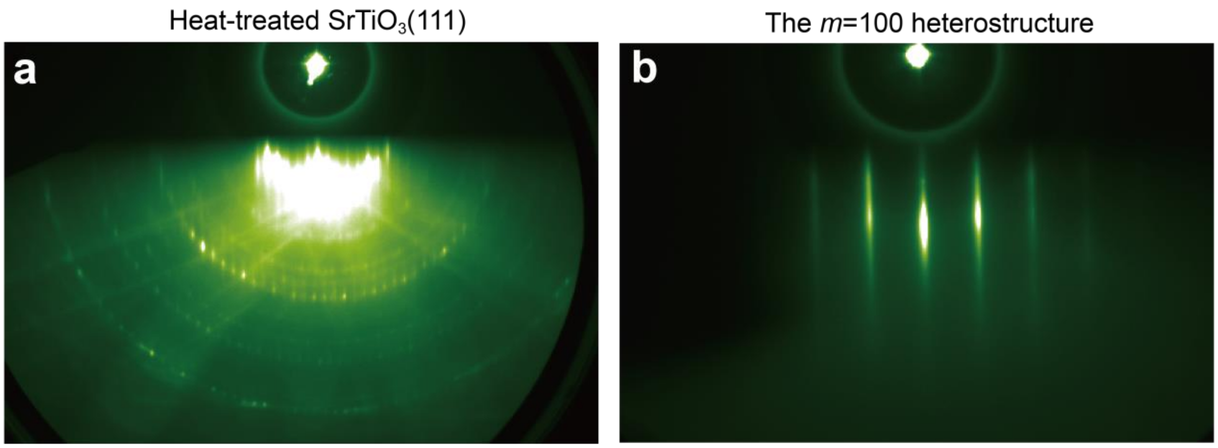

Supplementary Fig. 1| RHEED patterns of the *m*=100 sample. a, Heated-treated $SrTiO_3$(111) substrate. **b,** 3 QL V-doped $(Bi,Sb)_2Te_3$/100 QL $(Bi,Sb)_2Te_3$/3 QL Cr-doped $(Bi,Sb)_2Te_3$ (i.e., the *m*=100 sample) on $SrTiO_3$(111).

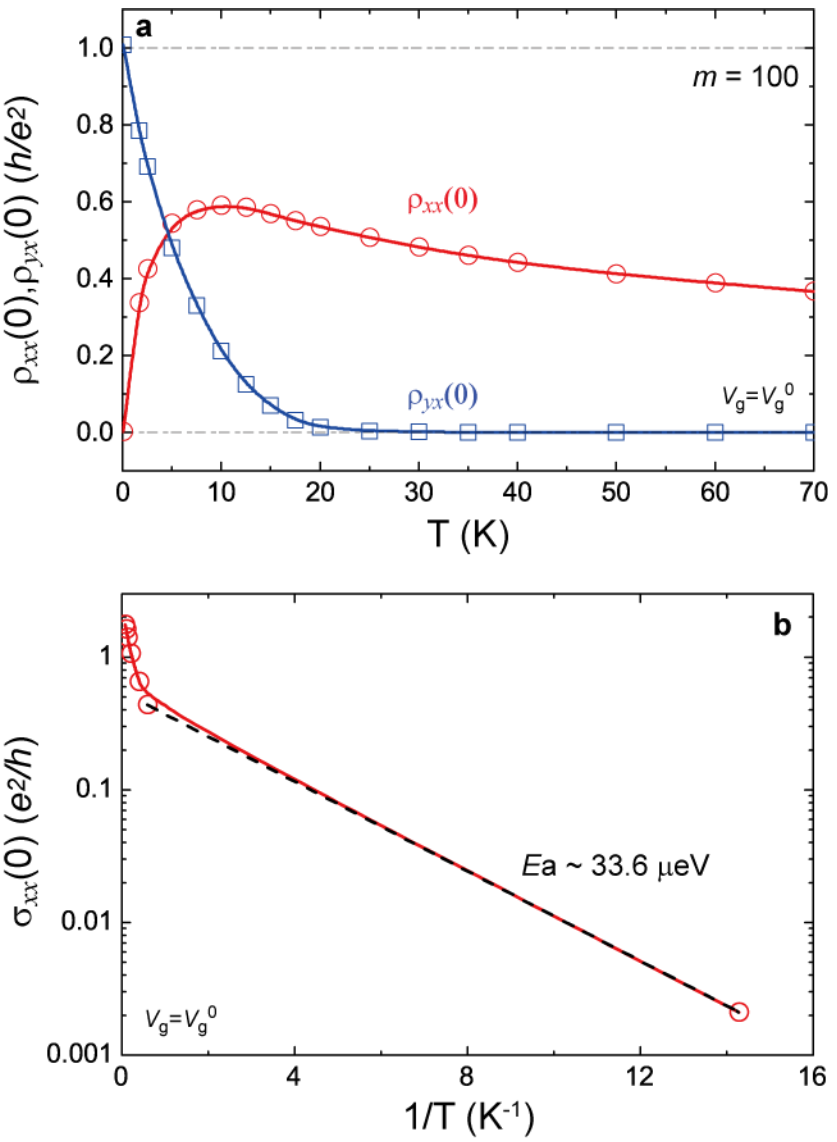

Supplementary Fig. 2| Temperature dependent ρ_{xx}(0) and ρ_{yx}(0) and estimate of the effective activation gap E_a in the *m*=100 sample. a, Temperature dependent ρ_{xx}(0) and ρ_{yx}(0) of the *m*=100 sample. The critical temperature of the QAH state is ~4.5 K. **b,** σ_{xx}(0) as a function of 1/*T*. The dashed lines show the fit of the Arrhenius function $\sigma_{xx} = \sigma_{xx}^0 e^{-\frac{E_a}{k_B T}}$. The fit temperature range is 0.07~1.7 K. All measurements are performed at $\mu_0 H$ = 0 T and $V_g = V_g^0$ after magnetic training. The estimated value of E_a is ~33.6μeV for the *m*=100 sample. This further supports our theoretical analysis that the gap of the side surfaces in our thick magnetic TI sandwiches is small.

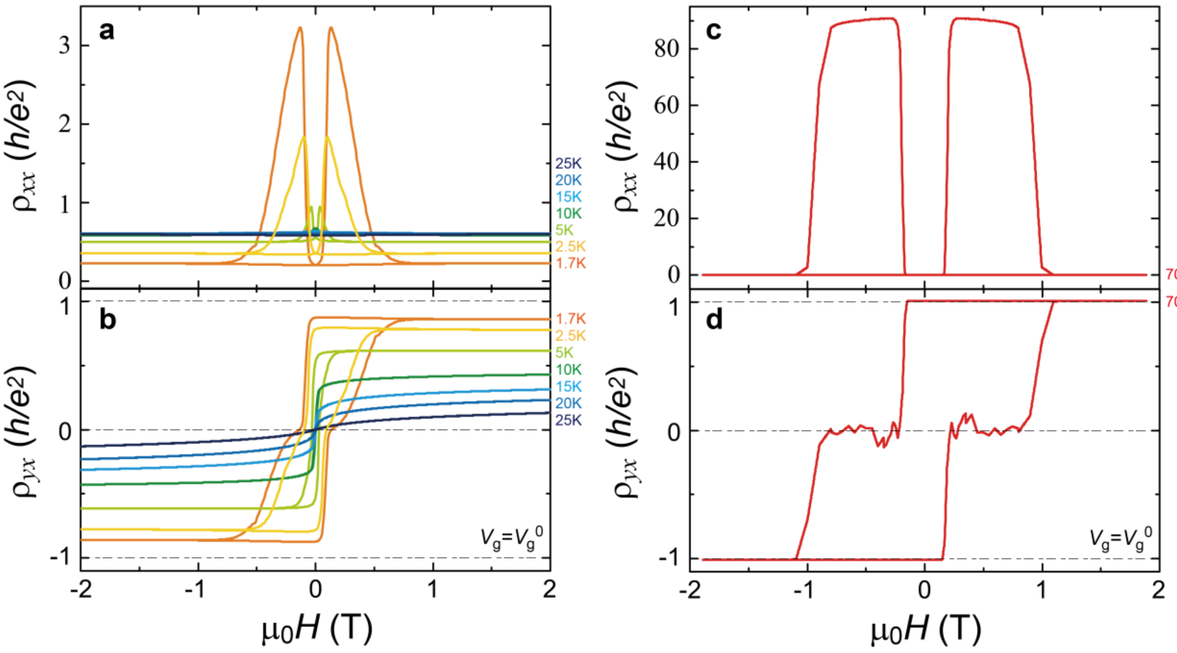

Supplementary Fig. 3| Magneto-transport results of the *m*=75 sample. a, b, $\mu_0 H$ dependence of ρ_{xx} (**a**) and ρ_{yx} (**b**) measured at different temperatures and $V_g = V_g^0$. The value of the Curie temperature T_C is ~15 K. **c**, **d**, $\mu_0 H$ dependence of ρ_{xx} (**c**) and ρ_{yx} (**d**) at T =70 mK and $V_g = V_g^0$.

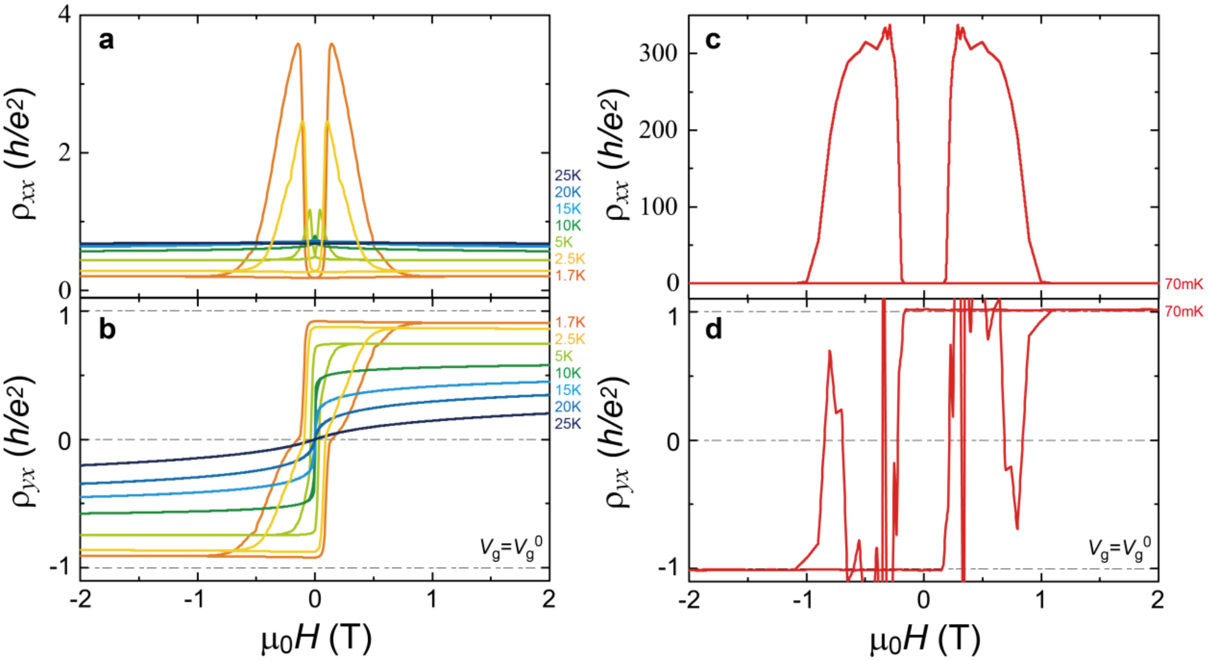

Supplementary Fig. 4| Magneto-transport results of the *m*=30 sample. a, b, μ_0H dependence of ρ_{xx} (**a**) and ρ_{yx} (**b**) measured at different temperatures and $V_g = V_g^0$. The value of the Curie temperature T_C is ~15 K. **c**, **d**, μ_0H dependence of ρ_{xx} (**c**) and ρ_{yx} (**d**) at T =70 mK and $V_g = V_g^0$.

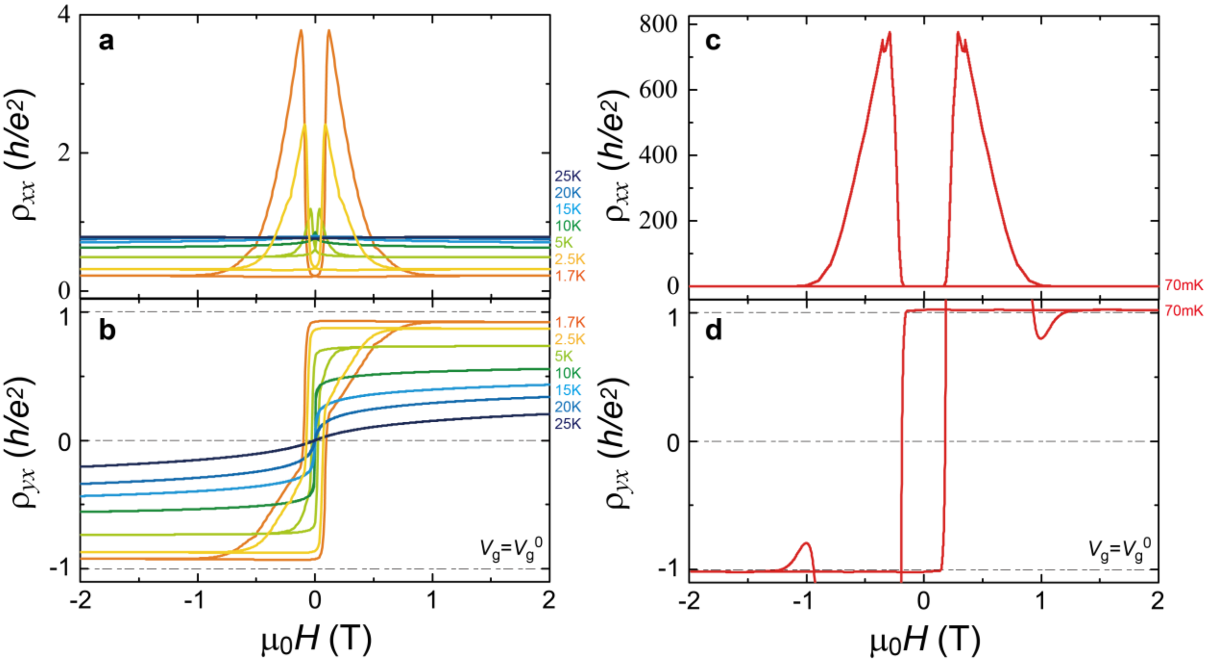

Supplementary Fig. 5| Magneto-transport results of the *m*=12 sample. a, b, $\mu_0 H$ dependence of ρ_{xx} (**a**) and ρ_{yx} (**b**) measured at different temperatures and $V_g = V_g^0$. The value of the Curie temperature T_C is ~15 K. **c**, **d**, $\mu_0 H$ dependence of ρ_{xx} (**c**) and ρ_{yx} (**d**) at T =70 mK and $V_g = V_g^0$.

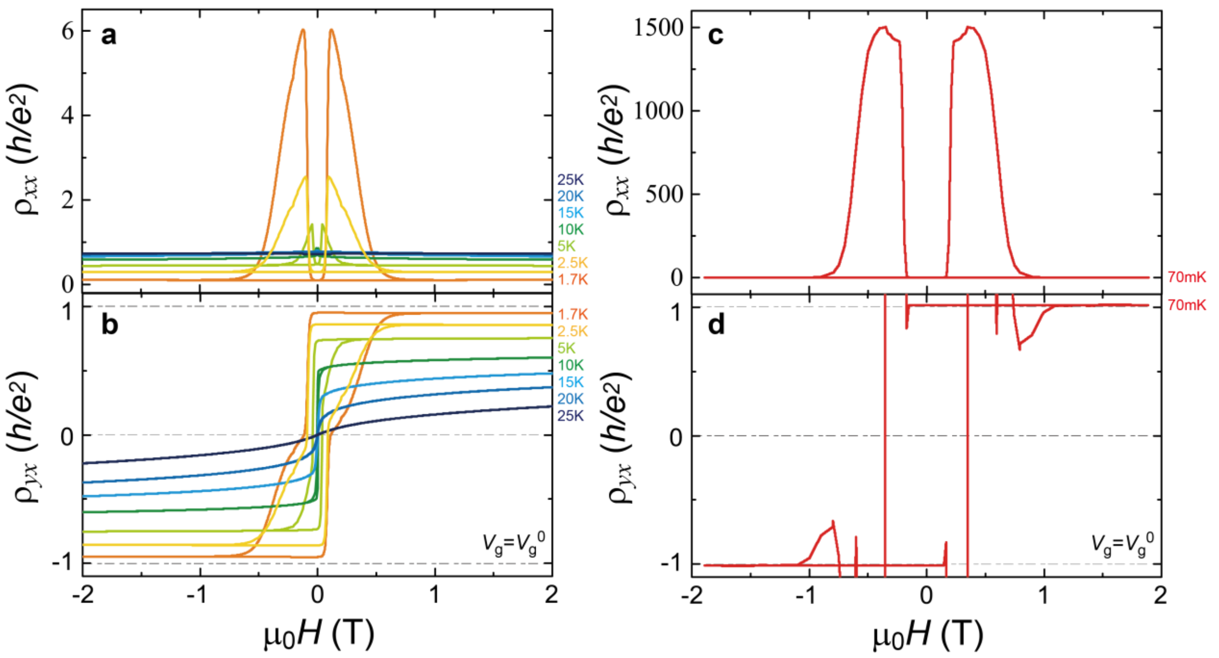

Supplementary Fig. 6| Magneto-transport results of the *m*=8 sample. a, b, $\mu_0 H$ dependence of ρ_{xx} (**a**) and ρ_{yx} (**b**) measured at different temperatures and $V_g = V_g^0$. The value of the Curie temperature T_C is ~15 K. **c**, **d**, $\mu_0 H$ dependence of ρ_{xx} (**c**) and ρ_{yx} (**d**) at T =70 mK and $V_g = V_g^0$.

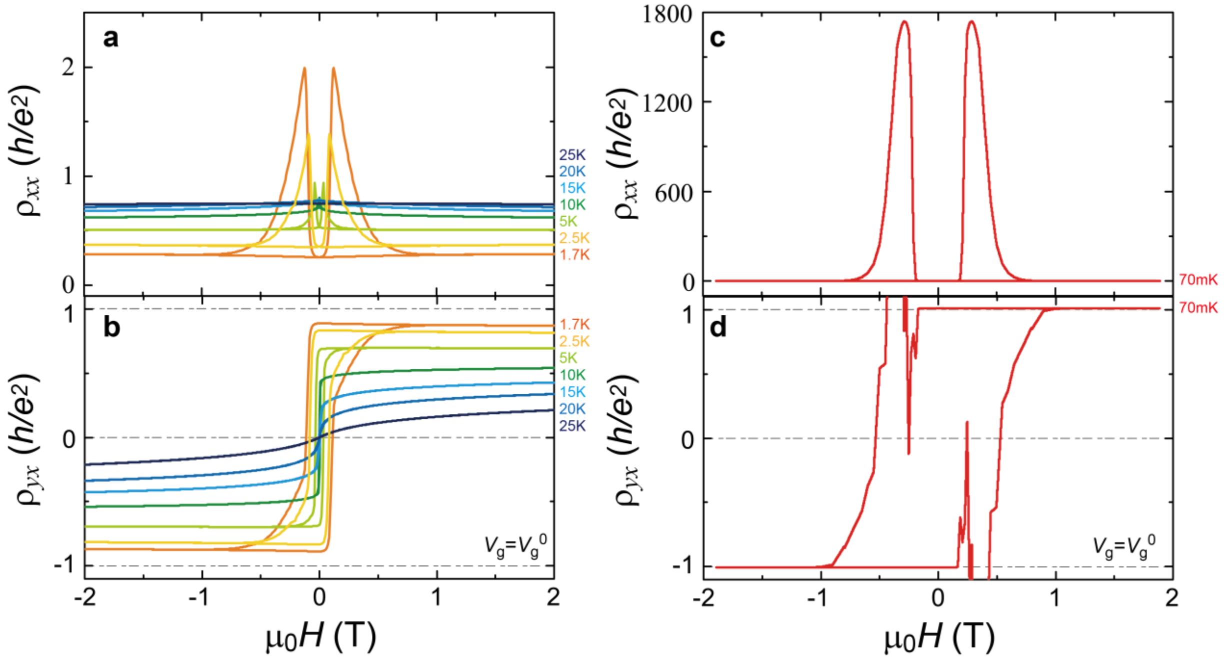

Supplementary Fig. 7| Magneto-transport results of the *m*=3 sample. a, b, $\mu_0 H$ dependence of ρ_{xx} (**a**) and ρ_{yx} (**b**) measured at different temperatures and $V_g = V_g^0$. The value of the Curie temperature T_C is ~15 K. **c**, **d**, $\mu_0 H$ dependence of ρ_{xx} (**c**) and ρ_{yx} (**d**) at T =70 mK and $V_g = V_g^0$.

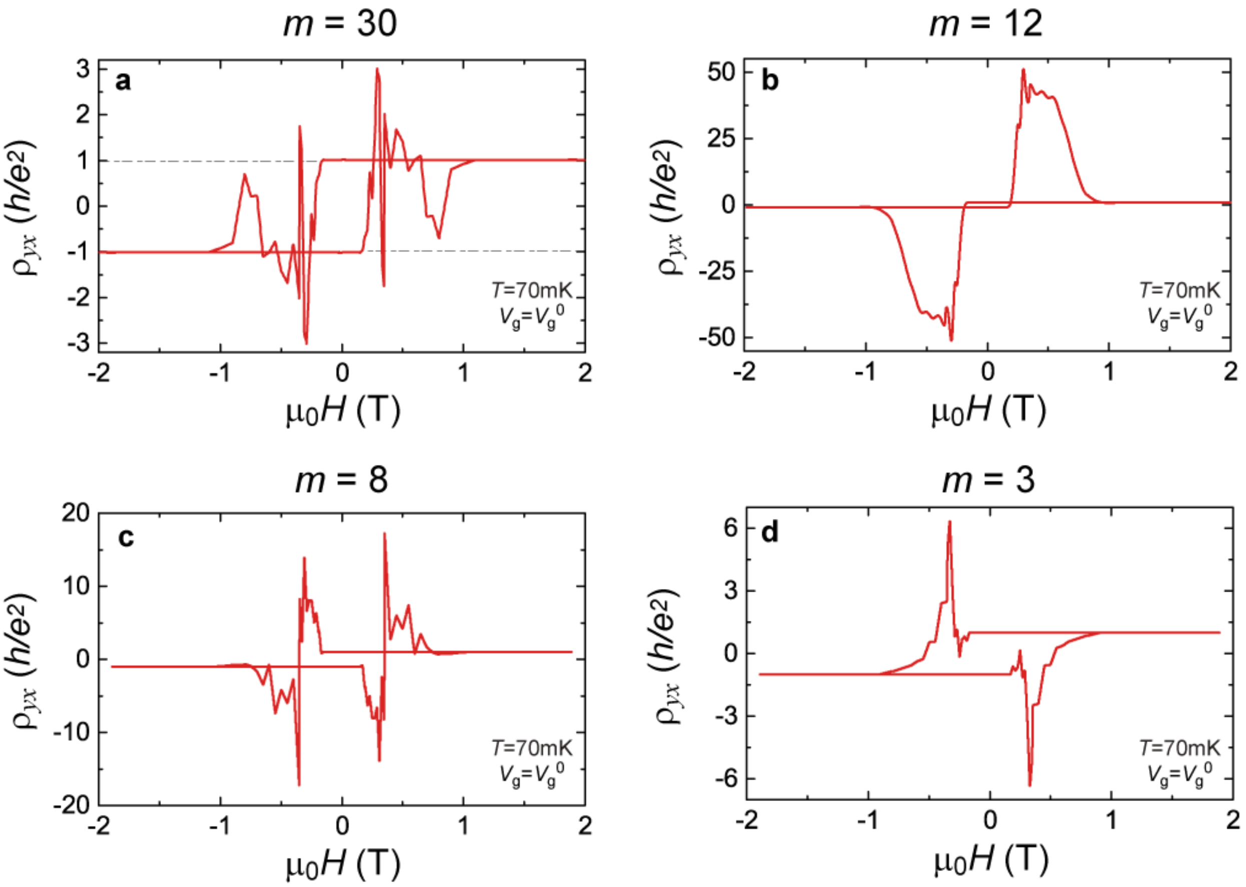

Supplementary Fig. 8| The full range of ρ_{yx}-$\mu_0 H$ curves in the $3 \leq m \leq 30$ samples. a-d, m=30 (**a**), m=12 (**b**), m=8 (**c**), and m=3 (**d**). All measurements are taken at T =70 mK and $V_g = V_g^0$.

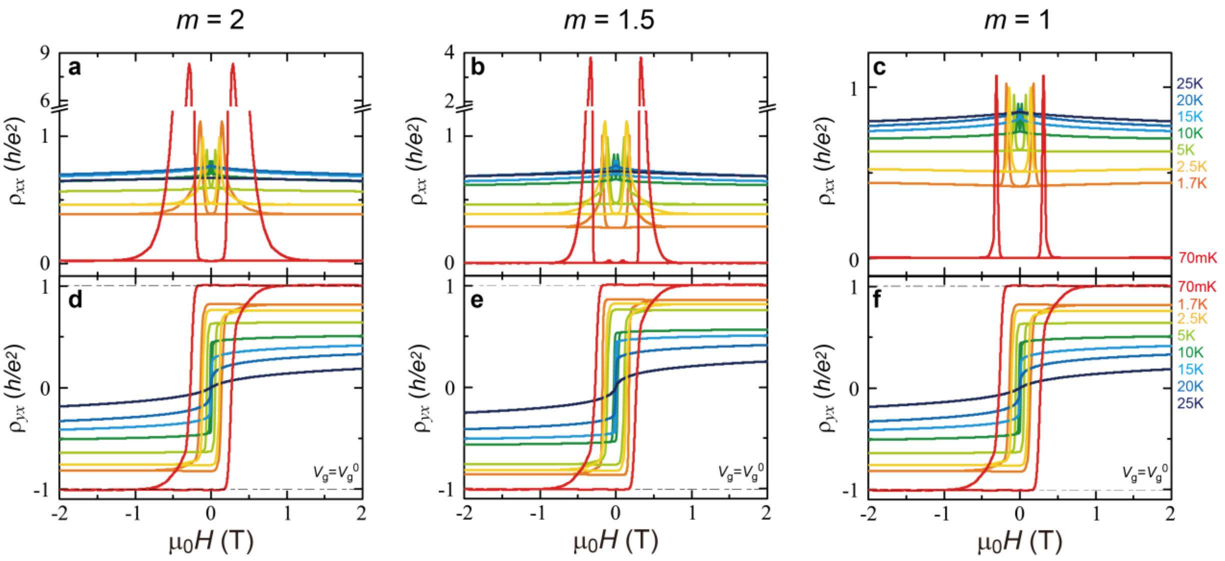

Supplementary Fig. 9| Magneto-transport results of the $m \leq 2$ samples. a-c, $\mu_0 H$ dependence of ρ_{xx} of the m=2 (**a**), m=1.5 (**b**), and m=1 (**c**) samples measured at different temperatures and $V_g = V_g^0$. **d-f,** Same as in **a-c**, but for ρ_{yx}. The values of the Curie temperature T_C are ~15 K, ~20 K, and ~20 K for the m=2, m=1.5, and m=1 samples, respectively.

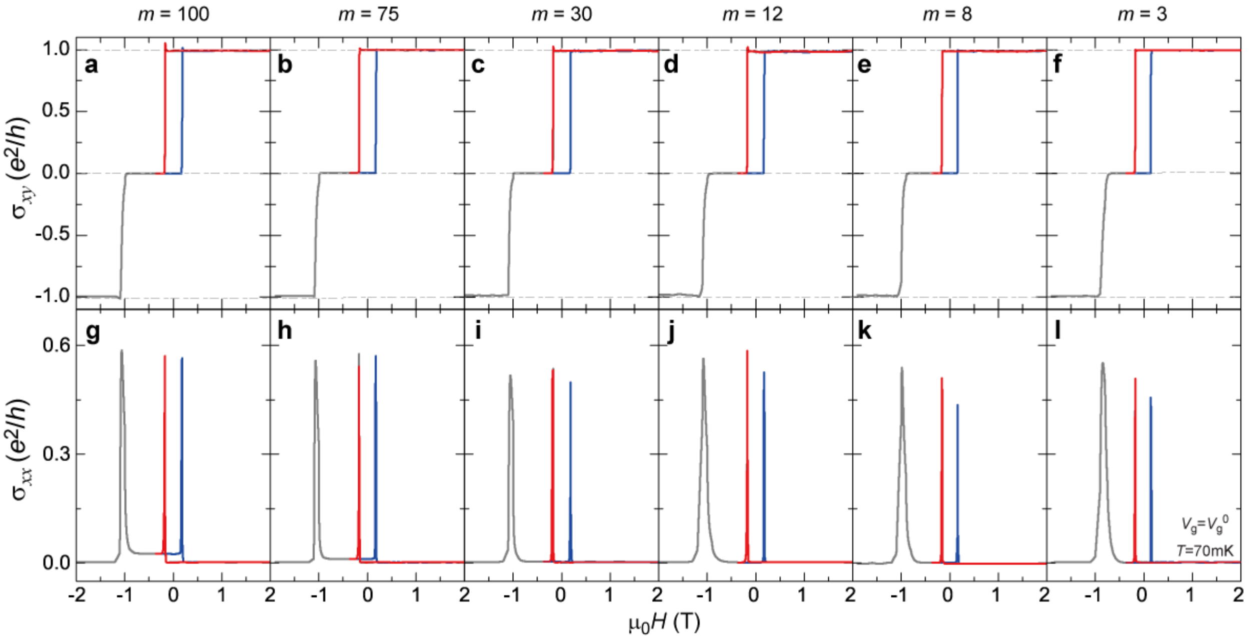

Supplementary Fig. 10| Minor loops of the *m* ≥ 3 samples. a-f, Minor loops of σ_{xy} of the magnetic TI sandwiches with *m*=100 (**a**), *m*=75 (**b**), *m*=30 (**c**), *m*=12 (**d**), *m*=8 (**e**), and *m*=3 (**f**), respectively. **g-l,** Same as in **a-f**, but for σ_{xx}. The red and blue curves represent sweeping $\mu_0 H$ downward and upward, respectively. The gray lines show the major loops of σ_{xy} and σ_{xx}. All measurements are performed at T =70 mK and $V_g = V_g^0$.

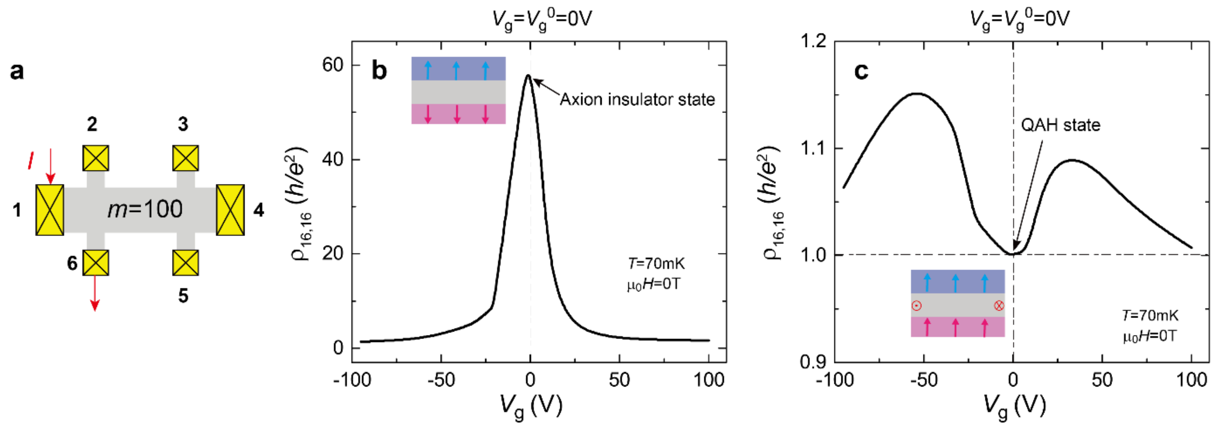

Supplementary Fig. 11| Gate dependence of the two-terminal resistance in the *m*=100 sample with antiparallel and parallel magnetization alignments. a, Schematics of the magnetic TI sandwich Hall bar device. The current flows from 1 to 6. **b, c,** V_g dependence of $\rho_{16,16}$ under antiparallel (**b**) and parallel (**c**) magnetization alignments. All measurements are taken at T =70 mK and $\mu_0 H$ =0T.

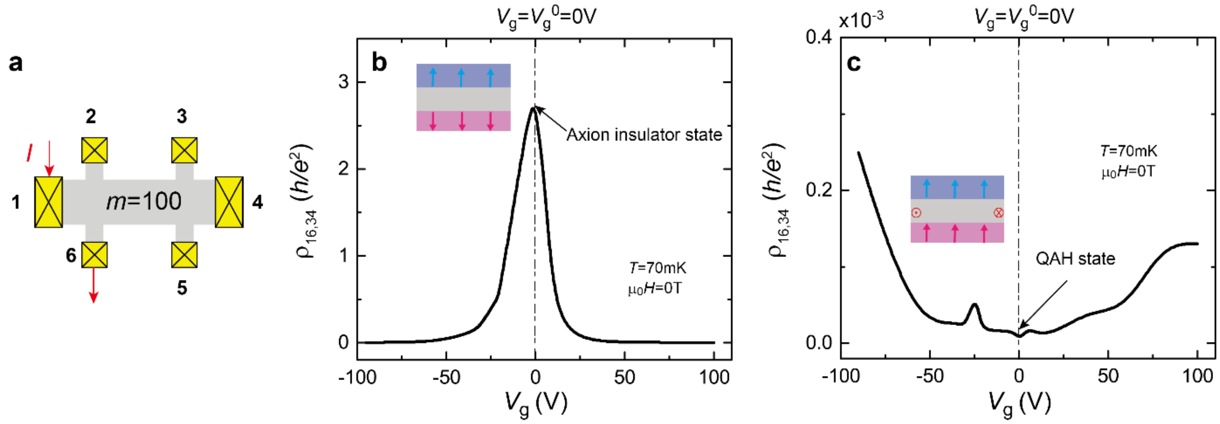

Supplementary Fig. 12| Gate dependence of the nonlocal resistance in the *m*=100 sample with antiparallel and parallel magnetization alignments. a, reused from **Supplementary Fig. 11a**. **b, c,** V_g dependence of $\rho_{16,34}$ under antiparallel (**b**) and parallel (**c**) magnetization alignments. All measurements are taken at T =70 mK and μ_0H =0T.

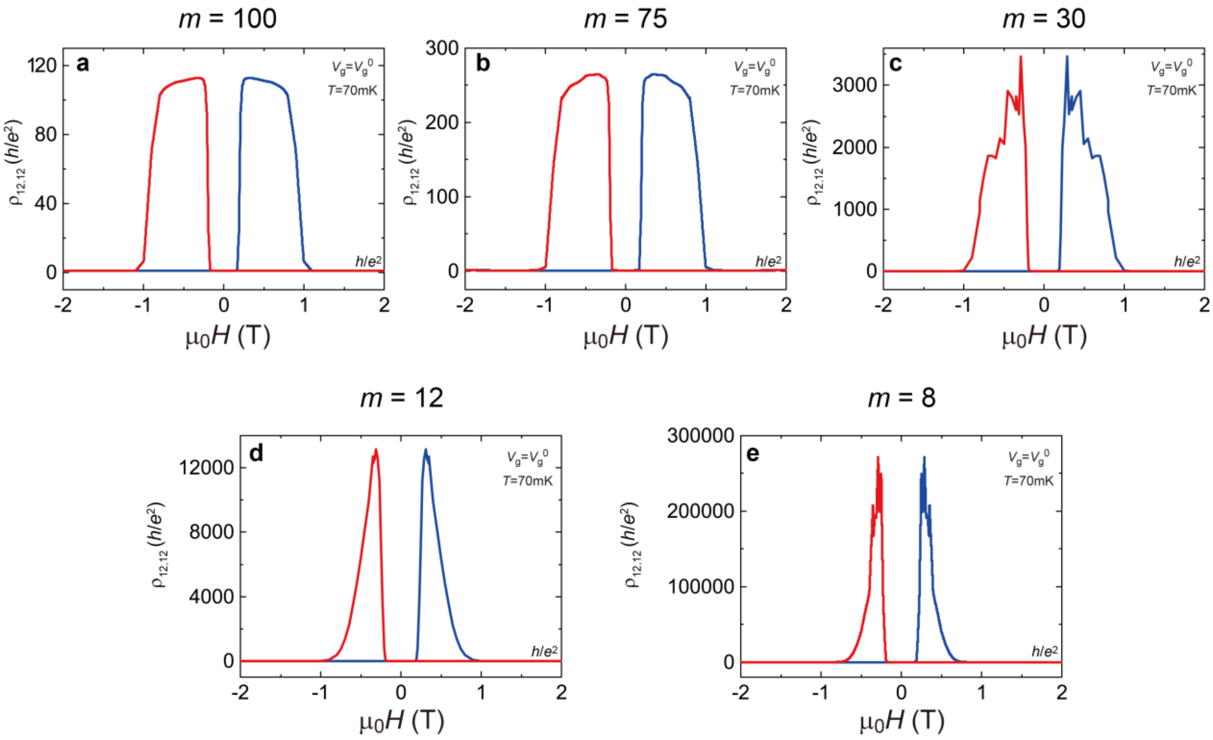

Supplementary Fig. 13| Two-terminal resistance $\rho_{12,12}$ of the $m \geq 8$ samples. a-e, $\mu_0 H$ dependent $\rho_{12,12}$ of the m=100 (**a**), m=75 (**b**), m=30 (**c**), m=12 (**d**), and m=8 (**e**) samples, respectively. The red and blue curves represent sweeping $\mu_0 H$ downward and upward, respectively. All measurements are performed at T =70 mK and $V_g = V_g^0$.

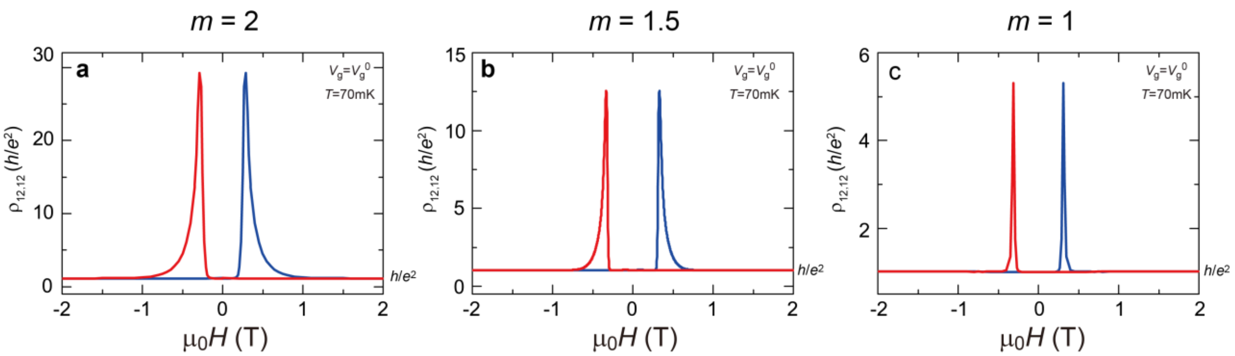

Supplementary Fig. 14| Two-terminal resistance $\rho_{12,12}$ of the $m \leq 2$ samples. a-c, $\mu_0 H$ dependent $\rho_{12,12}$ of the m=2 (**a**), m=1.5 (**b**), and m=1 (**c**) samples, respectively. The red and blue curves represent sweeping $\mu_0 H$ downward and upward, respectively. All measurements are performed at T =70 mK and $V_g = V_g^0$.

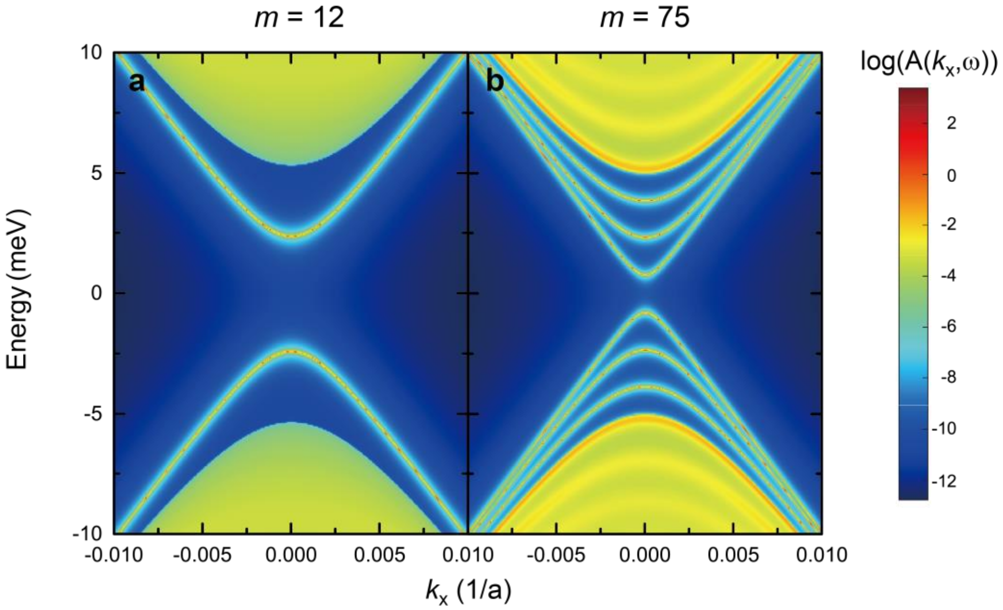

Supplementary Fig. 15| Quantum confinement-induced surface gaps in the *m*=12 and *m*=75 samples. a, b, Surface spectral functions in axion insulators with *m*=12 (**a**) and *m*=75 (**b**). 2*M* = 10 meV is used in (**a** and **b**).

II. Supplementary Text

1. Gate-dependent transport measurements on the *m*=100 sample

Besides the two-terminal measurements (Fig. 4 of the main text), we also measure V_g-dependent two-terminal resistance $\rho_{16,16}$ and nonlocal resistance $\rho_{16,34}$ of the m=100 sample when its top and bottom magnetic layers have antiparallel and parallel magnetization alignments (Supplementary Figs. 11 and 12). For the antiparallel magnetization alignment, $\rho_{16,16}$ shows a prominent peak at V_g= 0V and decreases dramatically when the chemical potential starts to cross the side surface bands (Supplementary Fig. 11b). However, for the parallel magnetization alignment, the value of $\rho_{16,16}$ is ~ h/e^2 near $V_g = V_g^0$ and does not change much when the chemical potential crosses the helical side surface bands[1].

We also perform nonlocal transport measurements on the m=100 sample (Supplementary Fig. 12). For the antiparallel magnetization alignment, the value of $\rho_{16,34}$ reaches its maximum at $V_g = V_g^0$ (i.e., the axion insulator phase). At first glance, this observation suggests the emergence of chiral edge currents in the axion insulator phase[2]. However, we would like to point out that the inherent “pickup” resulting from the extremely high ρ_{xx} value in the axion insulator state, together with the imperfect geometry of the Hall bar devices, can easily lead to a higher nonlocal signal.

2. The connection between the decay of the two-terminal resistance and the reduction of the transport gap

The connection between the decay of the two-terminal resistance and the reduction of the transport gap can be explained as follows: In the axion insulator regime of thick samples, the longitudinal transport is mainly determined by the side surface states, which represent the lowest energy states in the system. The side surfaces are gapped due to the finite size effect. For thinner samples, the side surface gap is large. On the other hand, in the 3D limit, the side surface states become metallic without a gap. Therefore, as m increases, there is a crossover from a fully gapped state to a metallic state. Figure 5e of the main text shows the side surface state gap as a function of m. Besides the numerical results, we also analytically show that the side surface gap

is proportional to m^{-1} for large *m*. The analytical results agree well with the numerical results.

Due to the side surface gap, the longitudinal resistance can be approximated by a formula for a thermally-activated semiconductor, which is proportional to $\exp(\delta/k_B T)$. Here δ represents the minimal gap in the side surface states and can be considered as the transport gap. As a result, the longitudinal resistance should decrease with increasing m as $\exp(K/k_B T m)$, with K being a constant. From this point of view, in the logarithmic scale, the resistance is also proportional to m^{-1}, which matches qualitatively with the experimental data well (Fig. 5e of the main text). Therefore, we suggest that the rapid decay of the longitudinal resistance with increasing *m* comes from the reduction of the side surface gap, which is inversely proportional to *m*.

3. Discussion on the thickness limit of the axion insulator state in magnetic TI sandwiches

We employ a phenomenological formula to estimate the two-terminal resistance $\rho_{12,12,max}$~ $K_1\exp(K_2/m)$ for large m. Through fitting the experimental data, we determine the values of K_1 and K_2 are ~$3.7h/e^2$ and 330, respectively. When the side surface gap δ is comparable with $k_B T$, the side surface should be metallic rather than insulating, and then the axion insulator state is no longer expected to be observed. The transition is given by the phenomenological formula, where K_2/m~1. Therefore, the critical thickness of the axion insulators is $m \approx K_2$=330.

Supplementary References

1 Chang, C. Z., Zhao, W. W., Kim, D. Y., Wei, P., Jain, J. K., Liu, C. X., Chan, M. H. W. & Moodera, J. S. Zero-Field Dissipationless Chiral Edge Transport and the Nature of Dissipation in the Quantum Anomalous Hall State. *Phys. Rev. Lett.* **115**, 057206 (2015).

2 Li, Y. X., Liu, C., Wang, Y. C., Lian, Z. C., Li, S., Li, H., Wu, Y., Lu, H. Z., Zhang, J. S. & Wang, Y. Y. Giant nonlocal edge conduction in the axion insulator state of MnBi2Te4. *Sci Bull* **68**, 1252-1258 (2023).
